# Automatic Crop Pest Detection Oriented Multiscale Feature Fusion Approach

**DOI:** 10.3390/insects13060554

**Published:** 2022-06-18

**Authors:** Shifeng Dong, Jianming Du, Lin Jiao, Fenmei Wang, Kang Liu, Yue Teng, Rujing Wang

**Affiliations:** 1 Institute of Intelligent Machines, Hefei Institutes of Physical Science, Chinese Academy of Sciences, Hefei 230031, China; dongsf@mail.ustc.edu.cn (S.D.); wfenmei@mail.ustc.edu.cn (F.W.); xinxill@mail.ustc.edu.cn (K.L.); yueteng@mail.ustc.edu.cn (Y.T.); rjwang@iim.ac.cn (R.W.); 2 Science Island Branch of Graduate School, University of Science and Technology of China, Hefei 230026, China; 3 School of Internet, Anhui Unviersity, Hefei 230039, China

**Keywords:** pest monitoring, deep learning, object detection, adaptive feature fusion

## Abstract

**Simple Summary:**

Monitoring pests is a labor-intensive and time-consuming task for agricultural experts. This paper proposes a new approach to classifying and counting different categories of crop pests. Specifically, we propose a multi-category pest detection network (MCPD-net), which includes a multiscale feature pyramid network and a novel adaptive feature region proposal network. The multiscale feature pyramid network is used to fuse the multiscale pest information, which significantly improves detection accuracy. The adaptive feature region proposal network addresses the problem of not aligning when region proposal network (RPN) iterating, especially for small pest objects. Extensive experiments on the multi-category pests dataset 2021 (MPD2021) demonstrated that the proposed method provides significant improvements in terms of average precision (AP) and average recall (AR); it outperformed other deep learning-based models.

**Abstract:**

Specialized pest control for agriculture is a high-priority agricultural issue. There are multiple categories of tiny pests, which pose significant challenges to monitoring. Previous work mainly relied on manual monitoring of pests, which was labor-intensive and time-consuming. Recently, deep-learning-based pest detection methods have achieved remarkable improvements and can be used for automatic pest monitoring. However, there are two main obstacles in the task of pest detection. (1) Small pests often go undetected because much information is lost during the network training process. (2) The highly similar physical appearances of some categories of pests make it difficult to distinguish the specific categories for networks. To alleviate the above problems, we proposed the multi-category pest detection network (MCPD-net), which includes a multiscale feature pyramid network (MFPN) and a novel adaptive feature region proposal network (AFRPN). MFPN can fuse the pest information in multiscale features, which significantly improves detection accuracy. AFRPN solves the problem of anchor and feature misalignment during RPN iterating, especially for small pest objects. In extensive experiments on the multi-category pests dataset 2021 (MPD2021), the proposed method achieved 67.3% mean average precision (mAP) and 89.3% average recall (AR), outperforming other deep learning-based models.

## 1. Introduction

Crop yields play an essential role in agricultural economic development. However, agricultural pests significantly affect crop production. Traditional pest recognition usually depends on manual observation by agricultural experts, whose work is subjective and labor-intensive [1,2,3]. Therefore, it is essential to propose a method that can automatically monitor pests and inform agricultural experts of the pest occurrence information timely. With the advancements in light trapping device technology, numerous pest images with high spatial resolution can be obtained easily, which makes automatic monitoring of different categories of pests possible. Therefore, using computer vision methods for pest recognition has become one of the hottest research topics.

Previous studies on pest recognition focused on conventional machine learning methods. They used image processing and machine learning approaches to extract pest features and then classify them. Gassoumi et al. [4] proposed a computer vision-based system to recognize pests of cotton. Boissard et al. [5] presented a cognitive vision system that combines knowledge-based techniques, image processing, and machine learning to detect mature whiteflies on rose leaves. Ebrahimi et al. [6] incorporated the image processing technique with the support vector machines (SVM) algorithm, successfully detecting thrips on the crop canopy images with an error rate of less than 2.5%. However, these methods are designed for classification based on manually designed features, which are time-consuming and inefficient, especially for multiple categories of pests. Therefore, a new technique with high accuracy should be presented for automatic pest detection.

Recently, some approaches based on deep convolution neural networks (DCNNs) have shown excellent performance on various vision tasks, including image classification, object detection, and object tracking. Object detection methods can be roughly divided into one-stage methods and two-stage methods. One-stage methods, such as the single shot multibox detector (SSD) [7], you only look once (YOLO) [8,9,10], RetinaNet [11], and the fully convolutional one-stage object detector (FCOS) [12], do not have a separate proposal generation stage. These methods usually consider all locations on the image as potential objects, and try to classify each region of interest as background or a target object. The typical two-stage methods include regions with convolutional neural network features (R-CNN) [13], Faster R-CNN [14], path aggregation feature pyramid networks (PAFPN) [15], and Mask R-CNN [16]. Those approaches divide the detection into two steps. The first step is region proposal generation, and the second step is classification of these region proposals. Although the above detection methods have superior performance on the common datasets, it is difficult to apply them directly to detecting pest images.

**Figure 1:**
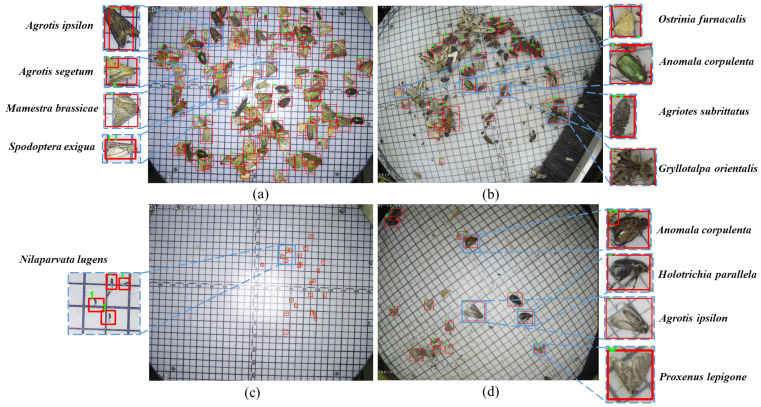
Examples of pest images in the MPD2021 dataset. (Ground truths indicated with the red bounding boxes). (**a**) Example of densely distributed pests. (**b**) Example of complex background image. (**c**) Example of tiny pests. (**d**) Example of normal pests.

The main limitations to its successful application are as follows: (1) The sizes of pests are diverse; some large pests are hundreds of times larger than small pests (Figure 1b,c). (2) Some categories of pests have physical similarities, and non-specialists are often unable to distinguish between species (Figure 1a,c). (3) Pest images contain multiple categories of pest objects that do not need to be detected, which makes the background complex (Figure 1b).

In this work, to address these problems, we propose a multi-category pest detection network (MCPD-net). First, a multiscale feature pyramid network (MFPN) for feature extraction is designed to obtain different scale information of pests. To obtain an enhanced feature pyramid network, an adaptive channel fusion module (ACFM) and a global context module (GCM) are introduced to capture recognizable multiscale contextual information to improve the detection accuracy for pests with diverse sizes, especially small pests. Second, an adaptive feature region proposal network (AFRPN) is proposed to obtain richer features with detailed information of pests. To alleviate the disturbances from complex backgrounds, a feature adaptation module (FAM) and a two-stage RPN method are introduced to correctly locate and classify pests. Third, we created a large-scale dataset named the multi-category pests dataset 2021 (MPD2021), containing 18,595 crop pest images with 26 categories and 125,700 specimens. Finally, with the MPD2021 dataset, extensive comparison experiments showed that our new model achieves 67.3% mean average precision (mAP) and 89.3% average recall (AR), which significantly outperforms other state-of-the-art methods.

To sum up, this work makes the following contributions:

(1) A large-scale dataset named MPD2021 was built, which will promote the effectiveness of applications of new object detection approaches in intelligent agriculture.

(2) A end-to-end detection method named MCPD-net is presented to detect large-scale pest images. The MFPN in MCPD-net can handle pests of various sizes, which significantly enhances the detection performance for multiscale pests.

(3) The presented AFRPN is able to solve the problem of anchor and feature inconsistency during the training iteration process, which benefits pest location and classification.

The rest of the paper is organized as follows: Section 2 describes the related works; Section 3 presents the pest image dataset description and analysis; Section 4 introduces the proposed method and technical details; Section 5 describes the experimental results; conclusions are covered in Section 6.

## 2. Related Works

### 2.1. CNN-Based Crop Pest Detection Method

Some DCNN-based approaches have been developed to solve the pest detection tasks [17,18,19,20,21,22]. Most of these pest detection methods are improvements on the object detection methods. To improve the detection performance, Deng et al. [18] detected ten categories of pests using the natural statistics model. It had strong recognition performance—an accuracy of 85.5%. Liu et al. [19] proposed PestNet, which can detect 16 categories of pests. It contains a channel-spatial attention (CSA) module used for feature enhancement and a position-sensitive score maps (PSSM) module to encode position information. Rustia et al. [20] proposed an online semi-supervised learning method and applied it to an automated insect pest monitoring system, thereby achieving a pseudo-labeling accuracy of 96.3%. To accurately detect tiny and densely distributed pests, Li et al. [17] developed a coarse convolutional neural network (CCNN) for searching aphid cliques and a fine convolutional neural network (FCNN) for refining the regions in aphid cliques, combined as a coarse-to-fine network (CFN) which detects tiny and densely distributed aphids. In recent work, Li et al. [21] presented a DCNN-based pests detection framework to classify ten categories of pests, which achieved excellent results. Wang et al. [22] developed a novel region proposal network (S-RPN) for generating accurate object proposals and a backbone network using an attention mechanism, which achieved 89.0% AR and 78.7% mAP on 21 categories of pests. These methods improve the detection accuracy of small pests through data augmentation strategies or enhanced network structure. However, the aspect of enhanced feature fusion has not been considered, which is necessary for the detection of pests with high inter-category similarity.

### 2.2. Feature Pyramid Network

FPN-based object detectors fuse multiscale features in a top-down and lateral connection manner to build a feature pyramid [23,24,25]. They have achieved great success on general datasets—e.g., MS COCO [26] and PASCAL VOC [27]. To integrate the balanced feature information from each resolution, Libra R-CNN [28] proposed a balanced feature pyramid (BFP) that integrates multi-level features using lateral connections and then refines balanced semantic features to reduce the imbalance between feature maps. EfficientDet [29] developed a BiFPN allowing efficient, bidirectional cross-scale connections. Some recent works have focused on adaptive feature fusion to improve the FPN’s ability. Adaptive spatial feature fusion (ASFF) [30] predicts the feature map’s weight factor from different layers during feature fusion via a self-adaptive mechanism. AugFPN [31] narrows the semantic gaps between features of different scales through consistent supervision. However, the aforementioned feature pyramid models mostly use the weighted fusion of upper and lower features and do not consider the channel-wise and global context view, which contains useful information. We introduced this information by using ACFM and GCM in MFPN.

### 2.3. Region Proposal Network

Region proposal networks (RPN) are frequently used in two-stage detectors. They are used to generate a sparse set of proposal boxes by adjusting the anchors. Traditional methods adopt selective search (SS) [32] and EdgeBoxes [33] approaches to generate proposal boxes. In this process, the imbalance between the foreground and background is increased due to the dense sampling of anchor boxes, which requires huge computations and leads to performance degradation. To address these problems, Faster R-CNN [14] was used in RPN to replace SS for object proposal generation, which is then refined and classified by R-CNN. Based on this, some improved solutions were developed to enhance the RPN’s proposal box generation. Vu et al. [34] proposed multi-stage refinement of the anchor box in each position, followed by using adaptive convolution to align the features and the anchors. In the dimension-decomposition region proposal network (DeRPN) [35], an anchor string mechanism is used to automatically match object shapes, which is less sensitive to variant object shapes. Besides, to avoid small objects being overwhelmed by larger objects, DeRPN [35] designed a novel scale-sensitive loss that addresses the problem of imbalanced loss computations for different scaled objects. As to improvement of the RPN to address the pest detection tasks, recently, an end-to-end deep learning approach (PestNet) [19] directly used the RPN module to search for potential region proposals for objects. Karar et al. [36] proposed a mobile application that utilizes RPN as an object bounds predictor for detection and classification of crop pests. A channel recalibration feature pyramid network (CRA-Net) [37] proposed an adaptive anchor (AA) module used in the RPN iteration to effectively correct the mismatch between the anchor and ground truth boxes. Therefore, we introduce AFRPN, combining the advantages of the above methods through FAM and a two-stage RPN method to align features with anchor boxes.

## 3. Materials

### 3.1. Light Trapping Device for Pest Monitoring

**Figure 2:**
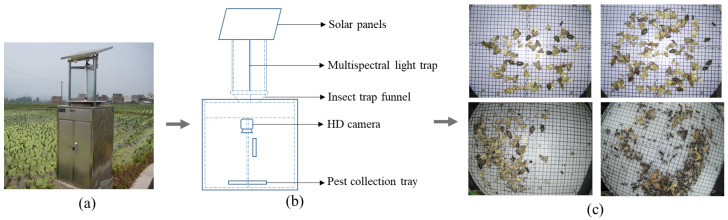
Pest image acquisition equipment. (**a**) Appearance of the device. (**b**) Inside structure. (**c**) Pest image examples.

The appearance and main internal structure of the light trapping device for pest monitoring are shown in Figure 2a,b. This equipment was designed by Jia Duo Co., Ltd. (Hebi, China) [38]. It can be placed in fields of vegetables, rice, corn, wheat and other major crops to monitor pests. This equipment has the functions of pest trapping and photography, environmental information collection, data transmission, data analysis, etc. In addition, using the proposed pest detection method can achieve pest classification and counting results. The statistical results are reported in real-time for automatic monitoring of pests. The multispectral light trap emits light to attract multiple categories of pests, the wavelength of which is changed with time according to pests’ habits. The collected pests are then dropped onto the collection plate at the bottom. Meanwhile, the HD camera above the tray is programmed to take photos every 15 min. The pests are swept away from the pest collection plate after being photographed to avoid gathering and overlapping. The collected images are saved with a resolution of 2592 × 1920 pixels. These pest images are sent to a cloud server, which recognizes the species and numbers of pests by a deep learning-based detection method.

### 3.2. Multi-Category Pest Dataset 2021 (MPD2021)

To promote the development of the field of automatic pest monitoring, some open datasets have been published so researchers can train models, such as IP102 [39] and the open access repository [40]. However, these datasets are mainly used for recognition, which does not meet the purpose of detecting multiple categories of pests in one image. In addition, the models trained using these datasets are also difficult to apply to pest images with complex scenes. To meet practical application requirements and train the detection model, a multi-category pest image dataset was built for pest detection tasks. Images of multiple categories of pests in the dataset were collected from a pest monitoring device. Each pest in the image was annotated as a bounding box using LabelImg software by several agriculture experts. Each bounding box has information about the upper left point, height, width, and pest category. The dataset was made in PASCAL VOC data format (an image dataset that contains 20 categories of objects; all objects are classified and annotated) [27], which uses XML files to record the pest labeling information. In summary, 125,700 labeled pests in 26 categories were annotated from 18,595 images. Based on this, a new dataset named MPD2021 was created.

Table 1 shows the statistics for each pest species, including the number of specimens, the average relative scale, and the average width and height of the labeled boxes. The number of specimens per species is from 241 to 24,694, for *Spodoptera frugiperda* (category 26) and *Proxenus lepigone* (category 7), respectively. The average width of pests ranges from 37 to 215 pixels, and the average height ranges from 35 to 211 pixels. All categories have a relative scale of less than 0.9%; the smallest average relative scale is only 0.0282%. As the MPD2021 dataset has a large number of small objects with poor feature information, this will cause significant challenges for network localization and accurate recognition.

**Figure 3:**
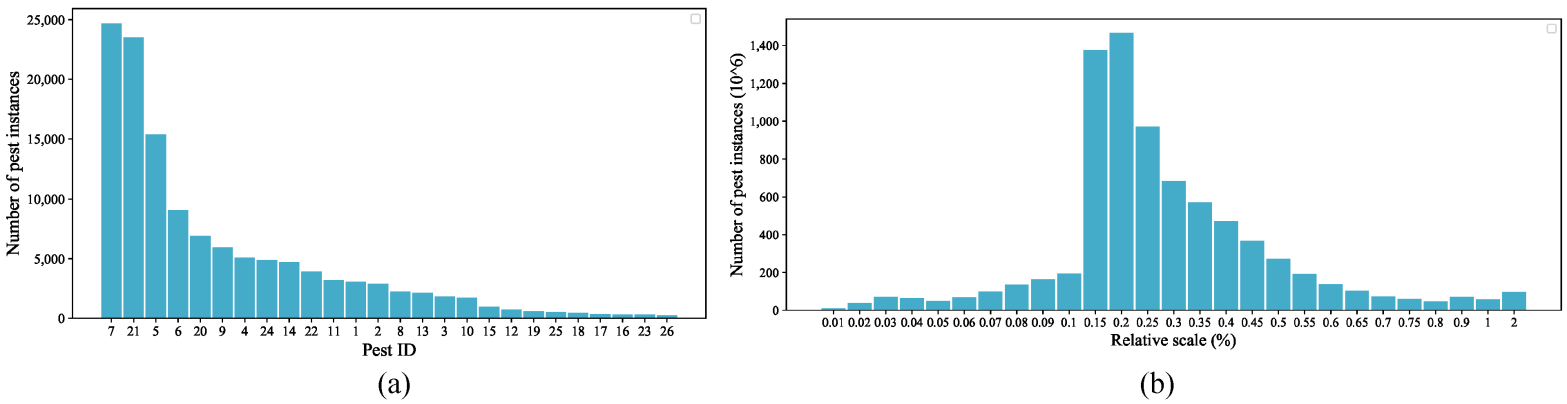
(**a**) Distribution of the number of pest specimens. (**b**) Relative scale distribution of the MPD2021 dataset.

To further analyzing the distribution of specimens in MPD2021, the distribution of specimens and relative scales are shown in Figure 3a. The distribution of the pest specimen numbers in the MPD2021 dataset varies greatly. The overall trend shows a long-tailed distribution for the number of specimens in each category, and the number of specimens in the most plentiful category is 102 times greater than that in the rarest category. For example, there are only 306 and 241 object specimens for *Pleonomus canaliculatus* (category 23) and *Spodoptera frugiperda* (category 26). To further analyze the scale problem, we analyzed the distribution of pest objects’ relative scale, as reported in Figure 3b. The relative size of the most pests in MPD2021 is comparatively small, mainly 0.15–0.5%. These collected pest images were randomly divided into a training set and a test set (4:1) to train the DCNNs models.

## 4. Proposed Method

### 4.1. MCPD-Net Construction

**Figure 4:**
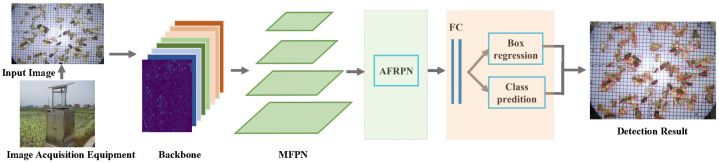
The technical pipeline of MCPD-net.

The overall architecture of MCPD-net is shown in Figure 4. We propose a unified framework named MCPD-net, which consists of four parts: (1) The pest images are first collected from the image acquisition equipment and then fed into the backbone networks. (2) A multiscale feature pyramid network (MFPN) for multiscale features with different spatial resolutions. (3) An adaptive feature region proposal network (AFRPN) for producing high-quality object proposals. (4) Two subnets for multi-category pest classification and box regression. The details are described in the following subsections.

### 4.2. Multiscale Feature Pyramid Network (MFPN)

**Figure 5:**
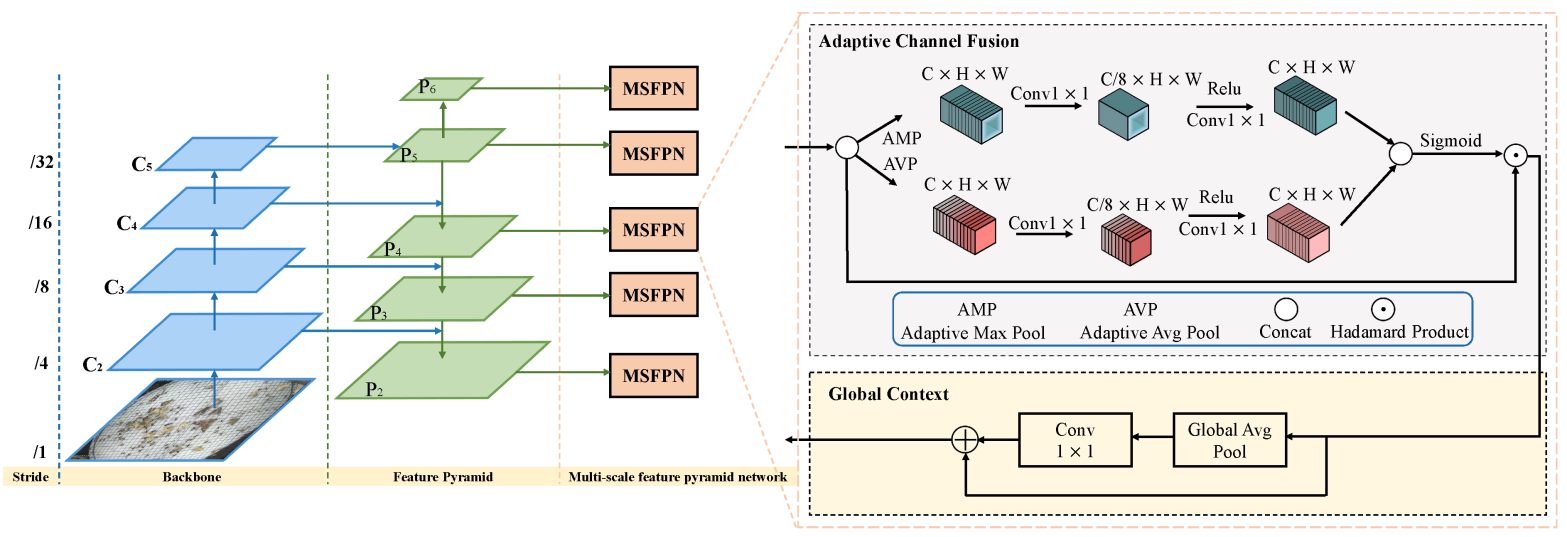
The network architecture of the proposed MFPN.

Some researchers developed detection approaches to address the challenges of multiscale features with different spatial resolutions. Following the setting of FPN [23], features used to build feature pyramid are denoted as $\left\{C_{2},C_{3},C_{4},C_{5}\right\}$, which correspond to the feature maps with upsampling {4,8,16,32} strides of the input images. Feature maps of $\left\{P_{2},P_{3},P_{4},P_{5}\right\}$ construct the feature pyramid networks. On the one hand, the low-level feature maps are enhanced by the high levels of semantic information; thus, the features will have diverse context information. On the other hand, there will be information loss from $C\in \left[C_{2},C_{3},C_{4},C_{5}\right]$ to $P\in \left[P_{2},P_{3},P_{4},P_{{}_{5}}\right]$ because of the reduction in feature channels and the decrease in the scale of the feature map, which leads to global semantic information loss. Pest images contain many small specimens that feature information usually suppresses due to complex background information and other large specimens. To address the problem of poor accuracy when detecting small objects, the typical approach only obtains the spatial information of multiscale feature maps to enhance the accuracy of small object detection. We argue that the information between feature channels and the global context is also essential for small objects. Thus, we designed MFPN to achieve accurate detection of multiple categories of pests. The overall framework of MFPN is shown in Figure 5. Two components of MFPN are discussed in the following subsections.

**Adaptive Channel Fusion:** To appropriately achieve feature fusion, we fully leverage the relationships between the feature maps. Let $C_{i}$ represent the i-th $C\in \left[C_{2},C_{3},C_{4},C_{5}\right]$. We compress the $C_{i}$ spatial information into the channel descriptors. Specifically, the 1×1 convolutional kernel is used for the high-level feature map to make uniform the number of channels, which can be expressed as:

(1) $$g_{c}=\frac{1}{H\times W}\sum_{x=1}^{H}\sum_{y=1}^{W}F_{c}\left(x,y\right)$$

where $g_{c}$ denotes the channel feature descriptor obtained by compressing, $F_{c}\left(x,y\right)$ indicates each pixel point (x,y) in the feature map, and H×W are the spatial dimensions of the feature map. We compute the relationships across channels using adaptive global averaging pooling (AGAP) and adaptive max-pooling (AMP) for a given channel descriptor $g_{c}\in R{}^{H\times W\times C}$. The subsequent operations can be described as:

(2) $$A_{C}=\sigma \left(A_{1}\left(g_{c}\right)+A_{2}\left(g_{c}\right)\right)$$

where σ represents the sigmoid activation function. Firstly, we split the operation into two branches, one branch for feature plane $g_{c}\in R{}^{H\times W\times C}$ using AGAP for the high-level feature map $F_{1}\in R{}^{H\times W\times C}$ extracted, and the other branch for adaptive max-pooling for the feature plane $g_{c}\in R{}^{H\times W\times C}$ to obtain $F_{2}\in R{}^{H\times W\times C}$. Secondly, convolution with kernels implementing 1×1 operations is conducted on the obtained channel descriptor, and then we obtain channel descriptors ${F_{1}}^{\prime }=f_{1D}\left(F_{1}\right)\in R{}^{H\times W\times C/8}$ and ${F_{2}}^{\prime }=f_{1D}\left(F_{2}\right)\in R{}^{H\times W\times C/8}$. Thirdly, the two channel descriptors ${F_{1}}^{\prime }$ and ${F_{2}}^{\prime }$ are computed by the Relu activation function and Conv1D operations to get F1″=f1D′(Relu(F1′))∈H×W×C, F2″=f1D′(Relu(F2′))∈H×W×C, respectively. Finally, the two channel descriptors $F_{1}{}^{\prime \prime }$ and $F_{2}{}^{\prime \prime }$ are summed to get the final channel descriptors $A_{C}\in {}^{H\times W\times C}$. Then, $A_{C}$ is activated by the sigmoid function, and the hadamard product operation is performed with the original high-level feature map to obtain $M_{C}\in {}^{H\times W\times C}$. The whole computation process can be summarized as follows:

(3) A1=f1D′(Relu(f1D(AvgPool(yc))))

(4) A2=f1D′(Relu(f1D(MaxPool(yc))))

(5) $$M_{C}=Had\left(A_{c}g_{c}\right)$$

where $AvgPool(y_{c})$ and $MaxPool(y_{c})$ denote the AGAP and AMP operations for each input feature map. $f{}_{1D}$ and $f{}_{1D}^{\prime }$ denote the one-dimensional convolution operations with kernel sizes of 1 to decrease the number of channels and increase the number of channels, respectively. The function Relu denotes the rectified linear unit activation function. Had denotes hadamard product operation.

**Global Context:** Detection performance and stability can be improved by exploiting global context information. Therefore, a global context module is introduced to strengthen MFPN. This module is integrated after the ACFM, as shown in Figure 5. Specifically, the GAP operation is first employed to extract the global information and then integrate information across channels by 1×1 convolution. Finally, the output features are added to the main information stream.

### 4.3. Adaptive Feature Region Proposal Network (AFRPN)

The detection problem is formulated in Faster R-CNN as a two-step procedure. The RPN is first used to generate a sparse set of proposal boxes by adjusting a set of anchors. The proposal boxes generated by the RPN are then refined and classified by a regional CNN detector. RPN is designed to extract high-level features and predict proposals in an end-to-end way. For a feature map $F_{i}$ of size w×h, a group of anchor boxes is initialized uniformly over the corresponding image. Each anchor box ***a*** consists of a set of four-dimensional information $a=(a_{x},a_{y},a_{w},a_{h})$, where $\left(a_{x},a_{y}\right)$ denotes the center location of the anchor and $\left(a_{w},a_{h}\right)$ is the width and height. The regression branch will predict the transformation value σ from the anchor box ***a*** to the ground-truth box ***g***, as follows:

(6) $$\begin{array}{cc} \; & a_{x}^{}=\sigma_{x}a_{w}+a_{x},a_{y}^{}=\sigma_{y}a_{h}+a_{y},\\ \; & a_{w}^{}=a_{w}\exp\left(\sigma_{w}\right),a_{h}^{}=a_{h}\exp\left(\sigma_{h}\right). \end{array}$$

**Figure 6:**
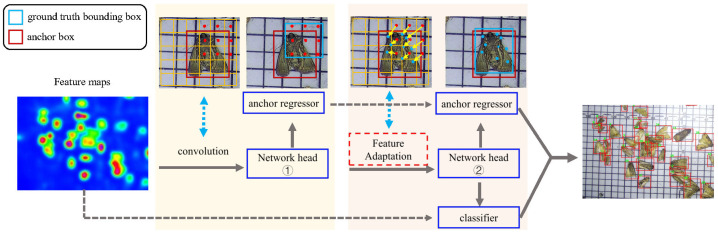
Illustration of AFRPN.

The regressed anchors A={a} are then filtered by non-maximum suppression (NMS) [41] to generate the sparse proposal boxes. However, in traditional RPN, each group of anchor boxes with different scales and aspect ratios is selected for positive samples based on the intersection of union (IoU) threshold with the label object. In this process, for small objects, the IoU values are usually too small to reach the set threshold. Therefore, most small object samples will be ignored as negative samples during the training process. Additionally, the anchor dense sampling will promote the imbalance between foreground and background, leading to module performance degradation. We propose an approach called AFRPN to systematically solve the aforementioned problem produced from the anchors and align features with anchor boxes. The pipeline of AFRPN is shown in Figure 6. During training iteration, AFRPN uses the conventional convolution to maintain the spatial features in the first stage and then adapts the proposed FAM to compute the regression prediction in the second stage to achieve high performance.

**Feature Adaptation:** The previous method adopts standard two-dimensional convolution to sample the feature map ***F*** in a regular grid $C=\left\{\left(c_{x},c_{y}\right)\right\}$, and then sum up the samples with the weight $w_{c}$. The grid size is defined by the convolution kernel size and the dilation factor. For instance,

$$\begin{array}{c} C=\left[\begin{array}{ccc} \left(-1,-1\right) & \left(-1,0\right) & \left(-1,1\right)\\ \left(0,-1\right) & \left(0,0\right) & \left(0,1\right)\\ \left(1,-1\right) & \left(1,0\right) & \left(1,1\right) \end{array}\right] \end{array}$$

corresponds to kernel size 3×3 and a dilation factor of 1. Then have $y\left[l\right]=\sum_{c\in C}w[c]\cdot F\left[l+c\right]$ for each location ***l*** on the output feature ***y***. However, in AFRPN the offset field O is directly inferred from the deformable convolution [42] that replaces the regular grid C. The output feature ***y*** will be $y\left[l\right]=\sum_{o\in O}^{}w[o]\cdot F\left[l+o\right]$. By learning the offset, the deformable convolution improves the spatial sampling location and ensures alignment between the anchors and features.

**Two-stage RPN:** A two-stage process is proposed to align anchors to features in the RPN stage. That is, the conventional convolution is used to maintain the spatial features in the first stage. In the following stages, the offset $o^{\kappa }$ of input anchor $a^{\kappa }$ on the feature map is computed by FAM. Then the regression prediction $\gamma^{\kappa }=f^{\kappa }\left(x,o^{\kappa }\right)$ and regressed anchor $a^{\kappa +1}$ from $\gamma^{\kappa }$ are computed using Equation (6). In the end, the object scores are calculated by the classifier and then filtered by NMS processing to generate region proposals.

## 5. Results

This section has a brief description of the evaluation metrics, training parameters, experimental details, and results of experiments on the constructed MPD2021 dataset.

### 5.1. Evaluation Metrics and Parameter Settings

Several evaluation metrics are used to evaluate the effectiveness of different approaches in the multi-category pest dataset. Average precision (AP) and mAP are used as the main evaluation metrics. AP is the area bounded by the precision–recall curve. In addition, ${\mathrm{AP}}_{50}$ (IoU = 0.5), ${\mathrm{AP}}_{75}$ (IoU = 0.75), average recall (AR), ${\mathrm{AR}}_{50}$ (IoU = 0.5), number of models parameters, and FLOPs—auxiliary evaluation metrics—are used to demonstrate the ability of MCPD-net. The calculation formulas are as follows:

(7) $$\mathrm{Precision}=\frac{\mathrm{TP}}{\mathrm{TP}+\mathrm{FP}}$$

(8) $$\mathrm{Recall}=\frac{\mathrm{TP}}{\mathrm{TP}+\mathrm{FN}}$$

(9) $$\mathrm{AP}=\int_{0}^{1}P\left(R\right)\mathrm{dR}$$

where TP, FP, and FN denote true positives, false positives, and false negatives, respectively.

In MCPD-net, the backbone network is Resnet-50, which was pretrained on the ImageNet [43] dataset. The images were resized to 1333 × 800 pixels during the training and validating stages. Moreover, the model was optimized during the training phase using the stochastic gradient descent (SGD) method. Specifically, the learning rate was $2.5\times 10^{-3}$ for the first eight epochs and then decayed with step policy for the following epochs, and the momentum and the weight decay values were 0.9 and 0.0001, respectively. The batch size was 4. We applied the NMS with the IoU threshold of 0.5 per category during the validating and testing stage. The code was developed based on the MMDetection toolbox [44]. All experiments were run on Dell Precision T3630 workstations equipped with Intel Core I9 9900K CPU, NVIDIA RTX 2080Ti (24-GB memory) GPU, and the software environment was Ubuntu 18.04, CUDA10.1 and CuDNN7.6, python 3.7.

### 5.2. Quantitative Analysis

In this section, the performance of MCPD-net is first compared with those of the state-of-the-art object detectors. Then, extensive ablation experiments on the MPD2021 dataset are reported to validate the effectiveness of the proposed module in MCPD-net. The testset in the MPD2021 dataset contains 3719 pest images. Table 2 presents the comparison between the AP and AR of MCPD-net and other CNN models. MCPD-net yielded 38.3% mAP detection accuracy, which surpasses all compared detectors. Our proposed method outperformed the detection performances of SSD (one-stage) and FCOS (anchor-free), achieving 6.4% and 5.2% mAP improvements, respectively. Compared with PAFPN and Mask R-CNN (multi-stage), our method achieved 3.2% and 3.6% AP improvements, respectively. Given these results, our method has the best performance. Compared to other IoU thresholds, our method achieved 67.3% ${\mathrm{AP}}_{50}$ and 40.4% ${\mathrm{AP}}_{75}$, which are higher than those of other detection approaches. Additionally, our method had a 55.4% AR, which indicates that it is more precise in object localization.

There are significant differences in the results for specific categories, as shown in Table 3. Our proposed approach significantly outperformed other detection methods in most pest categories. *Nilaparvata lugens* (category 1) seemed to be the most difficult to detect and had the lowest AP, 28.8%. Almost all models could successfully detect *Gryllotalpa orientalis* (category 22) with 94.0% AP. This is because tiny pests make it more difficult to extract effective features than larger pests. Furthermore, about 12 categories had over 70% detection accuracy, and the accuracy of almost all pest categories increased by using MCPD-net. The detection results for small pests *Nilaparvata lugens*, *Plutella xylostella*, *Cnaphalocrocis medinalis*, and *Melanotus caudex* (categories: 1, 2, 12, 25) showed different degrees of improvement.

#### Ablation Experiments

**(a) Baseline setup:** The baseline network is driven by Faster R-CNN with the backbone ResNet-50. It can be seen in Table 4 that the Faster R-CNN can quickly detect pest images at 22 FPS. However, the detection performance for small pests was not satisfying. For example, the mAP of the *Nilaparvata lugens* (category 1) was only 16.5%.

**Figure 7:**
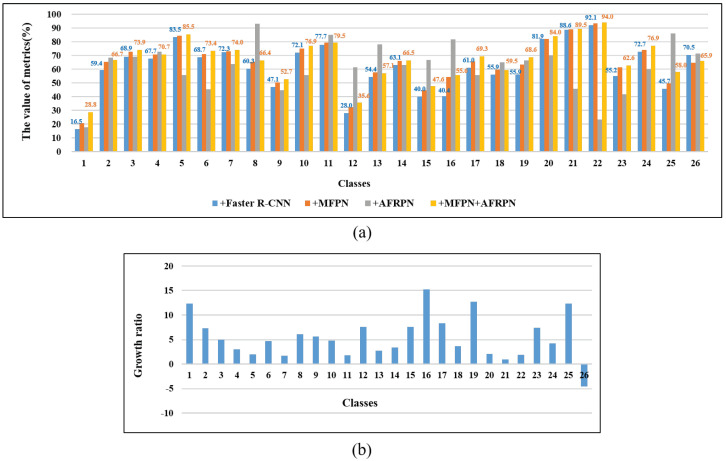
Detection results of our method on the MCPD2021 dataset. (**a**) Results of adding our proposed module. (**b**) Growth ratio of each category between our final result and baseline.

**(b) Effect of MFPN:** It can be seen in Table 4 and Figure 7 that the detection results were improved by adding the MFPN module. The detection AP of small pests showed significant improvements with the MFPN module. For instance, the detection results of tiny pests (categories 1 and 12) were improved by about 12.3% and 7.6% AP. The detection results of highly similar-in-appearance pests (categories 8, 11, 23) were also greatly enhanced. Additionally, our method achieved 64.9% ${\mathrm{AP}}_{50}$ and 80.7% ${\mathrm{AR}}_{50}$, which indicates that it is more precise in detection and more accurate at object localization. The detection results presented in Figure 7a were acquired by a multiscale structure from both high-level and low-level layer fusion, which proves that the MFPN is powerful.

**(c) Effect of AFRPN:** There are many easy negative samples during the training stage, which can lead to poor results. The AFRPN was proposed to solve this problem. As shown in Table 4, we achieved 87.8% ${\mathrm{AR}}_{50}$ after adding the AFRPN, which is a significant enhancement over the baseline. Figure 7 presents the efficiency of adding the AFRPN module, particularly concerning small pests. As a result, the overall detection accuracy slightly increased to 61.8% ${\mathrm{AP}}_{50}$. Finally, the whole detection framework (+ MFPN + AFRPN) can achieve the best pest detection result at 17 FPS. Although the inference speed is slightly slower than the baseline (22 FPS), the detection framework is suitable for accurate pest detection in real-world application scenes.

### 5.3. Visualization Analyses

**Figure 8:**
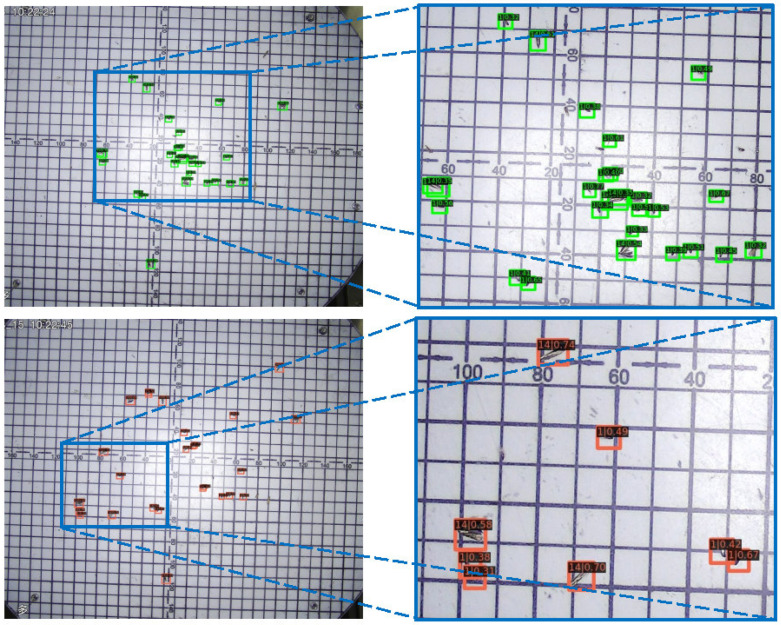
Detection results for tiny pests, *Nilaparvata lugens* (ID:1) and *Hadula trifolii* (ID:14).

**Figure 9:**
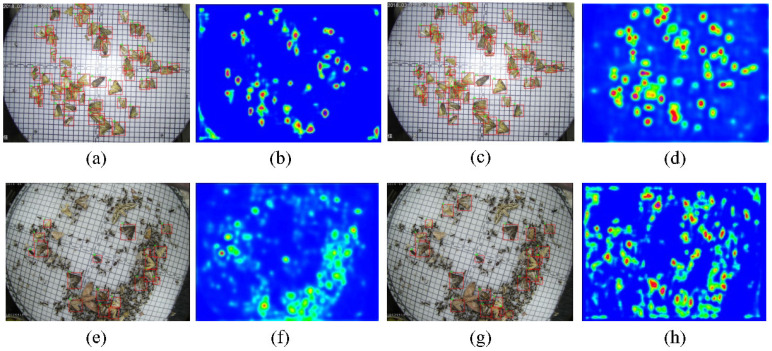
Visualization of detection results of different methods. (**a**,**b**) Results of the SSD method; (**c**,**d**) results of the proposed MCPD-net method; (**e**,**f**) results of the SSD method; (**g**,**h**) results of the proposed MCPD-net method.

Some visualization analyses were conducted to evaluate the proposed MCPD-net. As shown in Figure 8, MCPD-net has promising detection performance for tiny pests. Additionally, the detection and visualization results of different approaches on the MPD2021 dataset are shown in Figure 9. The visualizations of feature maps were obtained with the Grad-Cam [45] method. As presented in Figure 9a,b, the context information learned with the SSD method was not sufficient to accurately identify the pests. Figure 9c,d shows that MCPD-net obtains more pest feature information. Figure 9e,f shows the pest detection results in a complex background. As shown in Figure 9g,h, the contextual information of the pests is much richer, which indicates that the MCPD-net predicts more accurately and misses fewer pests.

**Figure 10:**
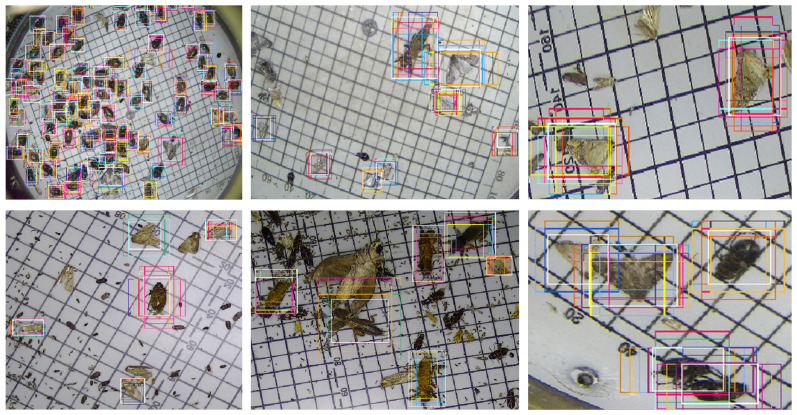
Illustration of the positive anchor samples (colored) in AFRPN and ground-truth boxes (white).

**Figure 11:**
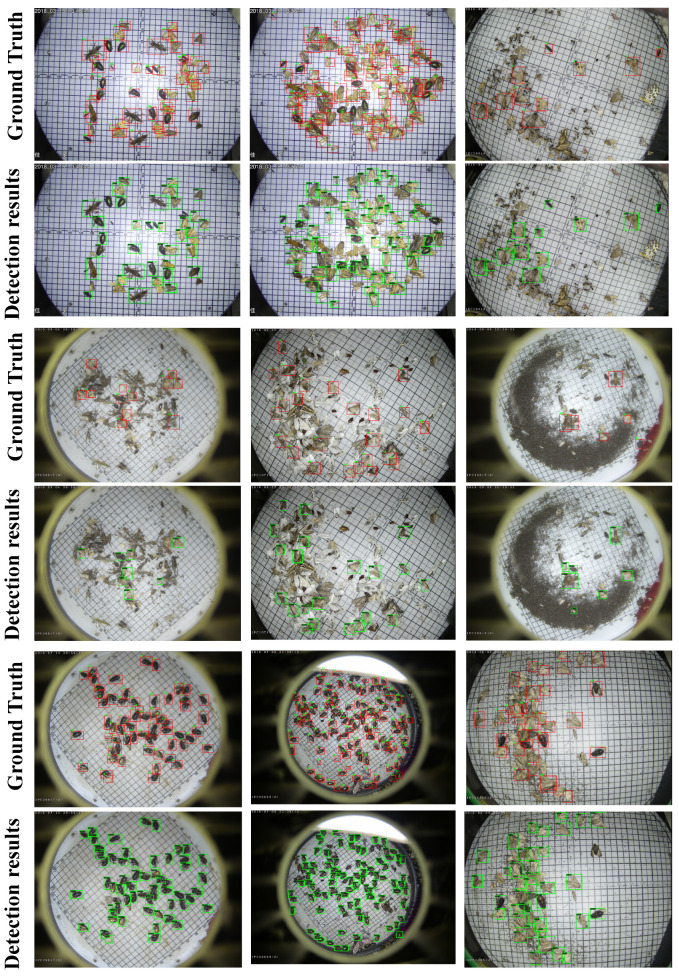
Detection results for each category on the MPD2021 dataset. Row 1, 3, and 5 are labeled boxes (red boxes); row 2 represents detection results of multi-category pest specimens per image; row 4 represents detection results in complex background images; row 6 represents the detection results of dense distribution images.

The positive anchor samples in AFRPN are illustrated in Figure 10. The anchor boxes (colored) can cover the ground-truth boxes (white) by the learning of AFM in AFRPN. In addition, the shapes of anchor boxes are close to those of the ground-truth boxes. This shows that our method improves the anchor prediction performance, so the localization capability is enhanced. The detection results for some typical images on the MPD2021 dataset are shown in Figure 11. Our method addresses the problems of a complex background, and small and dense pest distribution well. The detection results of rows 1, 3, and 5 are ground-truth boxes (red boxes). Row 2 of Figure 11 shows the detection results with multiple pest specimens in each image. MCPD-net can detect almost all of the labeled boxes. The detection results with complex backgrounds and dense pest distribution are shown in rows 4 and 6. The fine-grained information of multiple categories of pests is more distinct with our method, and the regression of pest boxes is more accurate. Therefore, we have demonstrated the generalization performance of MCPD-net for multi-category pest detection tasks on large-scale pest images.

### 5.4. Discussion

China is a large agricultural country. The main crops grown include rice, wheat, corn, etc. However, these crops are prone to reduced quality and yield of products due to crop pests. The widespread application of chemical pesticides has become an important means to prevent and control crop pests. However, farmers often tend to blindly use pesticides in large quantities when they cannot accurately identify pests, which can cause ecological environmental pollution and soil contamination. Thus, agricultural experts with professional knowledge are badly required to help them recognize pests. However, traditional pest recognition is usually labor-intensive and time-consuming. Intelligent light trapping devices can automatically attract many species of pests and capture images, which greatly reduces the workload of these experts. Although in practical applications, light trapping devices also attract trap pollinators and beneficial insects, the number of devices deployed is not large, so it will not have a huge impact on the environment. The collected images will be sent to a cloud server for analysis and monitoring. We proposed an object detection method named MCPD-net to automatically monitor multiple categories of pests to replace the manual observation method.

The proposed detection method can detect 26 categories of widespread agricultural pests (see Figure 1). When compared with other deep learning-based methods, the proposed MCPD-net achieved the highest detection accuracy, as shown in Table 3. The advantages of MCPD-net have been verified as follows. First, similar pests in images with complex backgrounds can be successfully detected. Second, MCPD-net is suitable for real-time detection of crop pests without prior knowledge of the acquired images about pest species. The proposed MFPN and AFRPN significantly improve the detection accuracy from the perspective of enhanced feature extraction, as shown in Figure 5 and Figure 6.

Our next research goal will be to further extend the number of pest categories in the dataset. This will support farmers to precisely apply chemical pesticides to protect the field from further damage. Although MCPD-net has achieved excellent experimental results, it still needs to be improved. For example, for *Nilaparvata lugens* (category 1) and *Plutella xylostella* (category 12), the detection results were only 28.8% AP and 35.6% AP, which are worse results than for other categories. They are too small, occupying only 0.0282% and 0.0545% pixels of the entire image. Our future work will aim to improve the detection accuracy for tiny pests.

## 6. Conclusions

In order to replace manual recognition methods with computer vision methods for automatic monitoring of pests in crop fields, we proposed a novel end-to-end method named MCPD-net that can be applied to detect 26 species of crop pests. MCPD-net consists of an MFPN for obtaining multiscale pest features, and the novel AFRPN makes the anchor box and features consistently. Extensive experiments were conducted on the MPD2021 dataset. MCPD-Net achieved 67.3% AP and 89.3% AR, surpassing other state-of-the-art methods.

## Figures and Tables

**Table 1:** The statistics of the MPD2021 dataset.

Pest ID	Scientific Names	Specimens	Average Width (Pixel)	Average Heigth (Pixel)	Relative Size (%)
1	*Nilaparvata lugens*	3045	37.8	35.4	0.0282
2	*Cnaphalocrocis medinalis*	2901	78.2	77.6	0.1223
3	*Chilo suppressalis*	1831	101.4	103.9	0.2086
4	*Mythimna separata*	5094	140.9	141.5	0.4122
5	*Helicoverpa armigera*	15,392	118.7	119.2	0.2945
6	*Ostrinia furnacalis*	9053	107.8	109.1	0.2400
7	*Proxenus lepigone*	24,694	83.8	85.0	0.1457
8	*Spodoptera litura*	2253	151.0	149.3	0.4523
9	*Spodoptera exigua*	5942	86.5	86.1	0.1497
10	*Sesamia inferens*	1740	123.0	123.1	0.3045
11	*Agrotis ipsilon*	3203	166.7	168.6	0.5768
12	*Plutella xylostella*	736	51.3	52.2	0.0545
13	*Mamestra brassicae*	2150	145.4	144.6	0.4300
14	*Hadula trifolii*	4725	130.9	130.8	0.3488
15	*Agrotis segetum*	981	145.4	145.5	0.4347
16	*Agrotis tokionis*	331	174.8	175.5	0.6269
17	*Agrotis exclamationis*	357	162.2	159.9	0.5192
18	*Xestia cnigrum*	446	140.9	139.4	0.4023
19	*Holotrichia oblita*	599	126.4	126.3	0.3195
20	*Holotrichia parallela*	6896	119.3	119.7	0.2900
21	*Anomala corpulenta*	23,523	109.2	109.6	0.2462
22	*Gryllotalpa orientalis*	3919	215.6	211.8	0.8993
23	*Pleonomus canaliculatus*	306	124.4	125.2	0.3230
24	*Agriotes subrittatus*	4893	80.7	82.2	0.1308
25	*Melanotus caudex*	532	74.6	74.0	0.1194
26	*Spodoptera frugiperda*	241	96.4	95.6	0.1886

**Table 2:** Different detection framework result (unit: %).

Method	Backbone	AP	${\mathrm{AP}}_{50}$	${\mathrm{AP}}_{75}$	AR
SSD(512)	VGG16	31.9	57.1	33.0	51.1
FCOS	ResNet-50	33.1	57.2	35.4	55.0
PAFPN	ResNet-50	35.1	61.5	37.2	49.8
Mask R-CNN	ResNet-50	34.7	60.9	36.4	49.9
Ours	ResNet-50	**38.3**	**67.3**	**40.4**	**55.4**

**Table 3:** ${\mathrm{AP}}_{50}$ of all pest categories with different detection methods (unit:%).

Pest ID	SSD 512 [7]	FCOS [12]	PAFPN [15]	Mask R-CNN [16]	Ours
1	5.3	8.9	16.1	15.3	**28.8**
2	49.6	55.3	57.0	59.4	**66.7**
3	60.6	66.6	68.1	67.6	**73.9**
4	62.3	67.3	67.1	66.3	**70.7**
5	82.5	**86.3**	83.5	84.1	85.5
6	64.5	70.4	68.7	68.2	**73.4**
7	67.7	73.0	72.0	72.0	**74.0**
8	56.0	60.9	61.9	59.9	**66.4**
9	44.0	45.8	47.8	47.5	**52.7**
10	67.8	70.8	71.4	70.4	**76.9**
11	74.6	78.4	77.1	78.0	**79.5**
12	12.6	14.0	29.5	27.4	**35.6**
13	43.8	54.0	54.9	54.3	**57.1**
14	56.8	61.6	63.3	63.2	**66.5**
15	40.1	23.9	42.3	40.4	**47.6**
16	45.2	25.9	42.6	40.8	**60.6**
17	**69.5**	60.5	63.2	62.5	69.3
18	51.8	47.5	55.5	54.6	**59.5**
19	51.2	63.9	55.7	52.6	**68.6**
20	**85.1**	83.8	82.0	81.4	84.0
21	**91.9**	90.8	88.7	88.4	89.5
22	93.7	93.5	92.6	93.2	**94.0**
23	54.0	58.5	53.0	53.5	**62.6**
24	71.1	73.7	72.7	73.4	**76.9**
25	44.2	4.8	42.1	44.1	**58.0**
26	56.4	59.7	**70.2**	65.9	65.9
AP	57.8	57.7	61.5	60.9	**67.3**

**Table 4:** Comparison of the performance with and without the proposed module.

Method	AP (%)	${\mathrm{AP}}_{50}$ (%)	${\mathrm{AR}}_{50}$ (%)	Params(M)	FLOPs(G)	FPS
Baseline	34.8	61.5	79.1	**41.24**	**206.78**	**22**
+MFPN	37.6	64.9	80.7	41.27	207.03	21
+AFRPN	35.5	61.8	87.8	41.53	206.84	18
+MFPN+AFRPN	**38.3**	**67.3**	**89.3**	42.12	207.70	17

## Data Availability

Not applicable.
